# Phylogenetic analysis of the Australian trans-Bass Strait millipede genus *Pogonosternum* (Carl, 1912) (Diplopoda, Polydesmida, Paradoxosomatidae) indicates multiple glacial refugia in southeastern Australia

**DOI:** 10.3897/zookeys.578.8052

**Published:** 2016-04-07

**Authors:** Peter Decker

**Affiliations:** 1 Senckenberg Museum of Natural History Görlitz, Am Museum 1, 02826 Görlitz, Germany

**Keywords:** Invertebrate, COI, 16S, 28S, genetic variability

## Abstract

This study documents the first detailed phylogenetic analysis of an Australian paradoxosomatid millipede genus. Two mitochondrial genes (partial COI and 16S) as well as partial nuclear 28S rDNA were amplified and sequenced for 41 individuals of the southeastern Australian genus *Pogonosternum* Jeekel, 1965. The analysis indicates that five species groups of *Pogonosternum* occur across New South Wales, Victoria and Tasmania: *Pogonosternum nigrovirgatum* (Carl, 1912), *Pogonosternum adrianae* Jeekel, 1982, *Pogonosternum laetificum* Jeekel, 1982 and two undescribed species. *Pogonosternum coniferum* (Jeekel, 1965) specimens cluster within *Pogonosternum nigrovirgatum*. Most of these five species groups exhibit a pattern of high intraspecific genetic variability and highly localized haplotypes, suggesting that they were confined to multiple Pleistocene refugia on the southeastern Australian mainland. The phylogenetic data also show that northwestern Tasmania was colonized by *Pogonosternum nigrovirgatum*, probably from central Victoria, and northeastern Tasmania by an as yet undescribed species from eastern Victoria.

## Introduction

*Pogonosternum* Jeekel, 1965 is the most widespread and species-rich genus of the millipede tribe Antichiropodini Brölemann, 1916 in Victoria, with the five described species *Pogonosternum nigrovirgatum* (Carl, 1902), *Pogonosternum coniferum* Jeekel, 1965, *Pogonosternum adrianae* Jeekel, 1982, *Pogonosternum laetificum* Jeekel, 1982 and the subspecies *Pogonosternum nigrovirgatum infuscum* Jeekel, 1982, all hitherto recorded from Victoria only. However, Jeekel (1982) and Mesibov and Churchill (2003) have recorded undescribed *Pogonosternum* species from Tasmania, and Car (2010) listed two undescribed *Pogonosternum* species from New South Wales.

Thus, *Pogonosternum* occurs on both sides of Bass Strait, which separates mainland Australia from Tasmania. The paradoxosomatid genus *Somethus* Chamberlin, 1920 also has a trans-Bass Strait distribution (Jeekel 2006), as do the paradoxosomatid species *Dicranogonus pix* Jeekel, 1982 and *Notodesmus scotius* Chamberlin, 1920 (Mesibov 2014).

Many soil invertebrates, including millipedes, have limited active dispersal capabilities. Phylogenetic studies of southeastern Australian soil invertebrates can give important insights into the impact of glacial periods during the Pleistocene (Byrne 2008, Endo et al. 2014, Garrick et al. 2004, Schultz et al. 2009, Sunnucks et al. 2006) and assist in identifying biogeographic barriers (Chapple et al. 2011). Unfortunately, phylogenetic studies of Australian millipedes are rare and restricted to a few taxa from a small number of localities (Adams and Humphreys 1993, Nistelberger et al. 2014, Wojcieszek and Simmons 2012). For the australiosomatine species *Orocladosoma kosciuskovagum* (Brölemann, 1913) from the Australian Alps a hypothesis of multiple glacial refugia has been proposed (Endo et al. 2014) to explain the results of such studies. Similarly, the australiosomatine genus *Somethus* in South Australia was found to have high morphological and genetic variability within species was discovered: it seems probable that isolation in multiple glacial refugia during the Pleistocene was the evolutionary driving force for this variability (Decker 2016).

The present study documents a molecular phylogenetic analysis of the antichiropodine genus *Pogonosternum*, using specimens from across the genus range, and with molecular evidence indicating past isolation in multiple Pleistocene refugia. Finally, the identity and origin of Tasmanian *Pogonosternum* populations are clarified.

## Material and methods

### Specimen collecting and preservation

**Figure 1.**
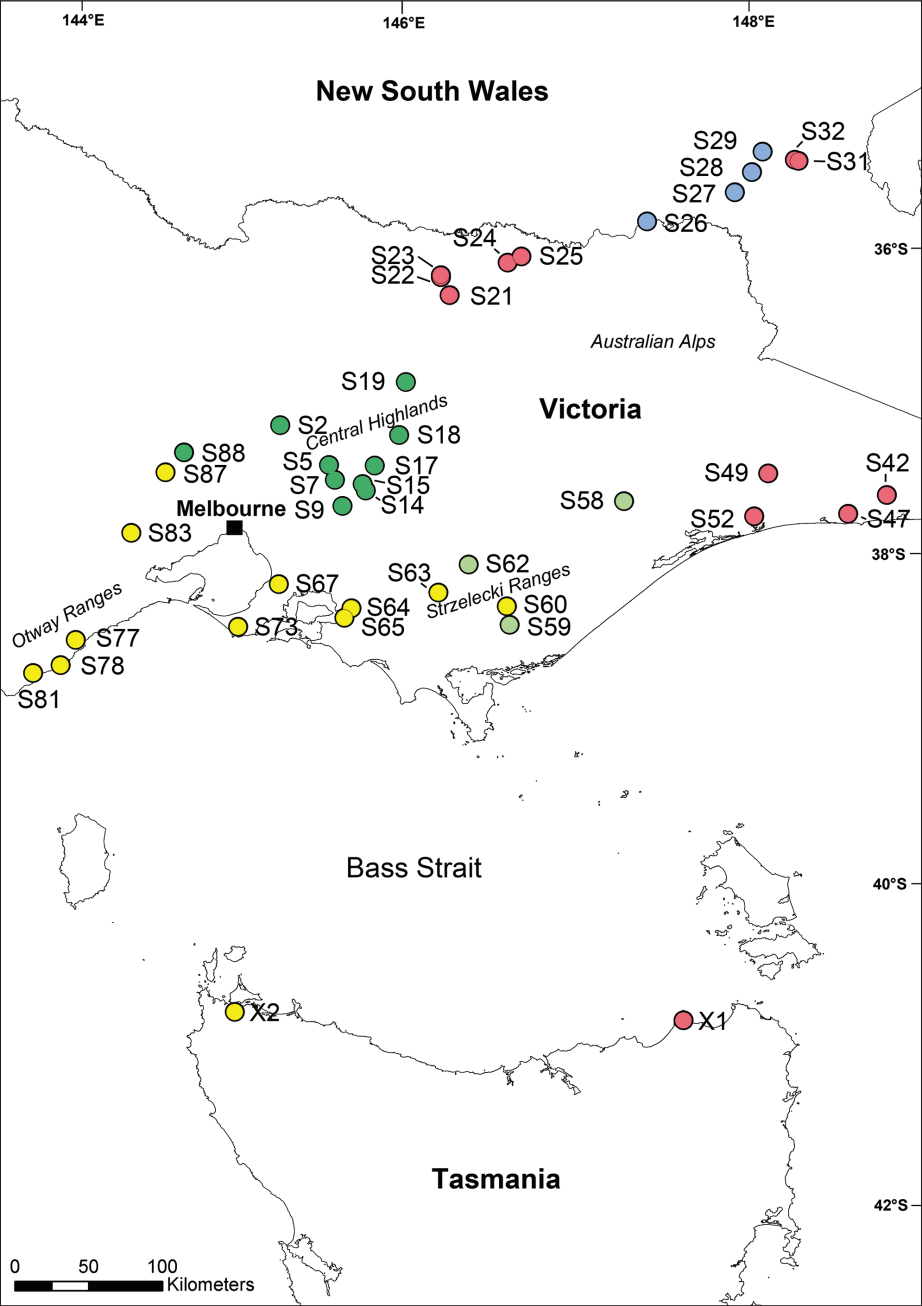
Map of Southeast Australia showing the distribution of *Pogonosternum* sampling sites with site numbers (see Table 1 and Suppl. material 1 for further details). *Pogonosternum adrianae* (light green), *Pogonosternum laetificum* (green), *Pogonosternum nigrovirgatum* s. l./*coniferum* (yellow), *Pogonosternum* sp. A (red), *Pogonosternum* sp. B (blue).

*Pogonosternum* specimens were collected by hand in Victoria and New South Wales in August 2014 by the author, Karin Voigtländer and Robert Mesibov, and by Mesibov in Tasmania in May 2014 and May 2015 (Fig. 1). Most sites were searched for 1-5 hours with the aim of finding 1-3 adult males. At only a few localities were *Pogonosternum* found to be abundant. Specimens were killed and stored in 95% ethanol, with a change of ethanol after 1–2 months. Full details of locality, date, collector, collection number and coordinates (WGS84 decimal degrees) are provided in Suppl. material 1.

**Table 1.** Site numbers, localities, GenBank accession numbers and repository accession numbers for all specimens analyzed. (See also Fig. 1) NMV = Museum Victoria, Melbourne, Victoria, Australia; QVMAG = Queen Victoria Museum and Art Gallery, Launceston, Tasmania, Australia; SAM = South Australian Museum, Adelaide, Australia; SMNG = Senckenberg Museum of Natural History Görlitz, Görlitz, Germany; NSW = New South Wales; SA = South Australia; TAS = Tasmania; VIC = Victoria. See Suppl. material 1 for further details.

Species	Site No.	Locality	GenBank Acc. No. COI	GenBank Acc. No. 16S	GenBank Acc. No. 28S	Voucher
**Outgroup**						
*Somethus scopiferus* Jeekel, 2002		SA, Martin Washpool Conservation Park	KT948674	KU833272		SMNG VNR016931
*Somethus castaneus* (Attems, 1944)		SA, Adelaide, Upper Sturt			KT964477	SAM OM2135
*Archicladosoma magnum* Jeekel, 1984		VIC, N Rawson	KT948681	KU833273		SMNG VNR016994
**Ingroup**						
*Pogonosternum adrianae*	S58	VIC, S Dargo	KU745235	KU745194	KU745185	NMV K-12203
*Pogonosternum adrianae*	S59	VIC, W Balook	KU745236	KU745195		NMV K-12204
*Pogonosternum adrianae*	S62	VIC, NE Moe	KU745237	KU745196	KU745186	NMV K-12207
*Pogonosternum coniferum*	S67	VIC, Langwarrin	KU745238	KU745197		NMV K-12212
*Pogonosternum coniferum*	S71	VIC, NE Cape Schanck	KU745239	KU745198		NMV K-12213
*Pogonosternum laetificum*	S2	VIC, NE Tyaak	KU745240	KU745199		NMV K-12095
*Pogonosternum laetificum*	S5	VIC, SE Glenburn	KU745241	KU745200		NMV K-12096
*Pogonosternum laetificum*	S7	VIC, E Toolangi	KU745242	KU745201		NMV K-12101
*Pogonosternum laetificum*	S9	VIC, SE Healesville	KU745243	KU745202		NMV K-12102
*Pogonosternum laetificum*	S14	VIC, SE Narbethong	KU745244	KU745203	KU745187	SMNG VNR016987
*Pogonosternum laetificum*	S15	VIC, E Narbethong	KU745245	KU745204		SMNG VNR016988
*Pogonosternum laetificum*	S17	VIC, N Marysville	KU745246	KU745205		NMV K-12109
*Pogonosternum laetificum*	S18	VIC, S Eildon	KU745247	KU745206		NMV K-12110
*Pogonosternum laetificum*	S19	VIC, W Barjarg	KU745248	KU745207		NMV K-12176
*Pogonosternum laetificum*	S88	VIC, Mt Macedon	KU745249	KU745208		NMV K-13113
*Pogonosternum nigrovirgatum*	S60	VIC, SE Traralgon South	KU745250	KU745209	KU745188	NMV K-12205
*Pogonosternum nigrovirgatum*	S63	VIC, SW Trafalgar	KU745251	KU745210		NMV K-12208
*Pogonosternum nigrovirgatum*	S64	VIC, W Nyora	KU745252	KU745211		SMNG VNR016989
*Pogonosternum nigrovirgatum*	S65	VIC, SE The Gurdies	KT948680	KU745212	KT964478	NMV K-12211
Pogonosternum cf. nigrovirgatum	S77	VIC, NW Lorne	KU745253	KU745213		SMNG VNR016990
Pogonosternum cf. nigrovirgatum	S78	VIC, W Kennett River	KU745254	KU745214		NMV K-13114
Pogonosternum cf. nigrovirgatum	S81	VIC, N Apollo Bay	KU745255	KU745215	KU745189	NMV K-13115
Pogonosternum cf. nigrovirgatum	S83	VIC, SW Staughton Vale	KU745256	KU745216		SMNG VNR016991
*Pogonosternum nigrovirgatum*	S87	VIC, W Gisborne	KU745257	KU745217		NMV K-13116
Pogonosternum cf. nigrovirgatum	X2	TAS, S West Montagu	KU745258	KU745218		QVMAG:2015:23:1
*Pogonosternum* sp. A	S21	VIC, N Glenrowan	KU745259	KU745219		NMV K-12177
*Pogonosternum* sp. A	S22	VIC, NE Thoona I	KU745260	KU745220		NMV K-12178
*Pogonosternum* sp. A	S23	VIC, NE Thoona II	KU745261	KU745221		NMV K-12179
*Pogonosternum* sp. A	S24	VIC, SE Chiltern	KU745262	KU745222		SMNG VNR016992
*Pogonosternum* sp. A	S25	VIC, SSW Chiltern	KU745263	KU745223	KU745190	NMV K-12181
*Pogonosternum* sp. A	S31	NSW, E Talbingo I	KU745264	KU745224		NMV K-12187
*Pogonosternum* sp. A	S32	NSW, E Talbingo II	KU745265	KU745225		NMV K-12188
*Pogonosternum* sp. A	S42	VIC, NNW Bemm River	KU745266	KU745226		NMV K-12192
*Pogonosternum* sp. A	S47	VIC, E Orbost	KU745267	KU745227		NMV K-12195
*Pogonosternum* sp. A	S49	VIC, Buchan	KU745268	KU745228		NMV K-12197
*Pogonosternum* sp. A	S52	VIC, SW Nowa Nowa	KU745269	KU745229		NMV K-12199
*Pogonosternum* sp. A	X1	TAS, W Tomahawk	KU745270	KU745230	KU745191	SMNG VNR016986
*Pogonosternum* sp. B	S26	NSW, SE Holbrook	KU745271	KU745231		NMV K-12182
*Pogonosternum* sp. B	S27	NSW, W Tumbarumba	KU745272	KU745232		NMV K-12183
*Pogonosternum* sp. B	S28	NSW, NNE Tumbarumba	KU745273	KU745233	KU745192	SMNG VNR016993
*Pogonosternum* sp. B	S29	NSW, SE Batlow	KU745274	KU745234	KU745193	NMV K-12185

### Illustrations

Maps were created with ArcGIS 10. The final phylogenetic trees were edited using Adobe Illustrator CS4.

### Molecular analysis

DNA was extracted from 2-4 legs from each of 41 *Pogonosternum* specimens and from the three paradoxosomatid species *Archicladosoma magnum* Jeekel, 1984, *Somethus scopiferus* Jeekel, 2002 and *Somethus castaneus* (Attems, 1944), which were chosen as outgroups (Table 1). Total genomic DNA was extracted using the Qiagen DNAeasy Blood&Tissue kit following the standard protocol except that tissue was incubated for 48h.

Glom primer cocktail pairs (Decker 2016, Macek et al. 2014) were used to sequence a 618 bp fragment of the mitochondrial cytochrome *c* oxidase subunit I (COI) gene. Primer pairs 28S D1a (Fw) and 28S D3b (Rv) (Dell’Ampio et al. 2009) were used to amplify 1225 bp of the D2 fragment and adjacent areas of D1 and D3 on the nuclear 28S ribosomal RNA gene.

For PCR protocol and all primer sequences (COI, 28S) see Decker (2016).

Primer pairs 16Sar (Fw) (5’-CGCCTGTTTAACAAAAACAT-3’) and 16Sbr (Rv) (5’-CCGGTCTGAACTCAGATCACGT-3’) (Simon et al. 1994) were used to sequence a 566 bp fragment of the large-subunit ribosomal RNA (16S) gene. The following thermocycling profile was used to amplify fragments of 16S: pre-denaturation at 94°C for 4 min 30 sec, 35 cycles of 30 sec at 94°C, 30 sec at 49°C and 50 sec at 72°C, and the final extension step for 5 min at 72°C.

All PCR mixes had a total volume of 10 µl comprising 1 µl template, 0.2 µM of each primer, 4x0.2 mM dNTPs [Peqlab], 1 x PCR Buffer containing 1.5 mM MgCl_2_ [Peqlab], and 0.05u Polymerase [Peqlab].

All fragments were sequenced in both directions by the BiK-F Laboratory Centre, Frankfurt, Germany. All obtained sequences were checked via BLAST searches of GenBank; no contamination was discovered. The sequences were aligned by hand in ClustalX ver. 1.83 (Chenna et al. 2003) and uploaded to GenBank (Table 1).

Some homologisation problems in the 16S rRNA sequences arose mainly because of the highly variable expansion loops. As a result, selected alignment positions (272-297) were excluded from the 16S rRNA dataset for all further analyses using MEGA6.

The final alignments consisted of 618 bp of COI mtDNA, 540 bp of 16S rRNA and 1206 bp of 28S rRNA. The combined datasets after these exclusions consisted of 1158 bp for COI+16S. Individual partial alignments can be obtained from the author upon request. The alignment of the combined dataset can be found in the Suppl. material 2 as a FASTA file.

COI and 16S sequences were combined as a single dataset and incongruence assessed between the mtDNA intergenic spacer sequences with the incongruence length difference (ILD) test (Farris et al. 1994) implemented as the partition homogeneity test in PAUP* version 4.0b10 using a full heuristic search, 10 random taxon addition replicates, tree-bisection-reconnection (TBR) branch swapping, and with MaxTrees set to 100 (Swofford 2002). The best-fit model of nucleotide substitution for the individual COI and 16S dataset was determined by MrModelTest 2 (Nylander 2004). The best-fit model of nucleotide substitution selected using MrModelTest 2 was the General Time Reversible model with gamma distribution and proportion of invariant sites (Nei and Kumar 2000) for the individual COI and 16S dataset. The trees constructed from individual genes did not show significant conflicts in topology (nodes different among trees with support > 70% in ML) and no significant incongruence among the three genes was revealed by the ILD test (*P* > 0.83 in all of the pairwise comparisons), so the sequences were concatenated into a dataset containing 1158 characters for phylogenetic analysis.

The combined dataset of COI and 16S was analysed under maximum likelihood (ML) using MEGA6 (Tamura et al. 2011) and Bayesian inference (BI) using MrBayes version 3.2 (Ronquist et al. 2012). For ML analysis, three independent runs were performed with nodal support estimated from 1000 bootstrap (BP) pseudoreplicates using the best-fit model for the concatenated dataset. For Bayesian analysis, two independent runs were carried out with four differentially heated Metropolis-coupled Monte Carlo Markov chains for 10 000 000 generations started from a random tree and chains were sampled every 100 generations.

**Figure 2.**
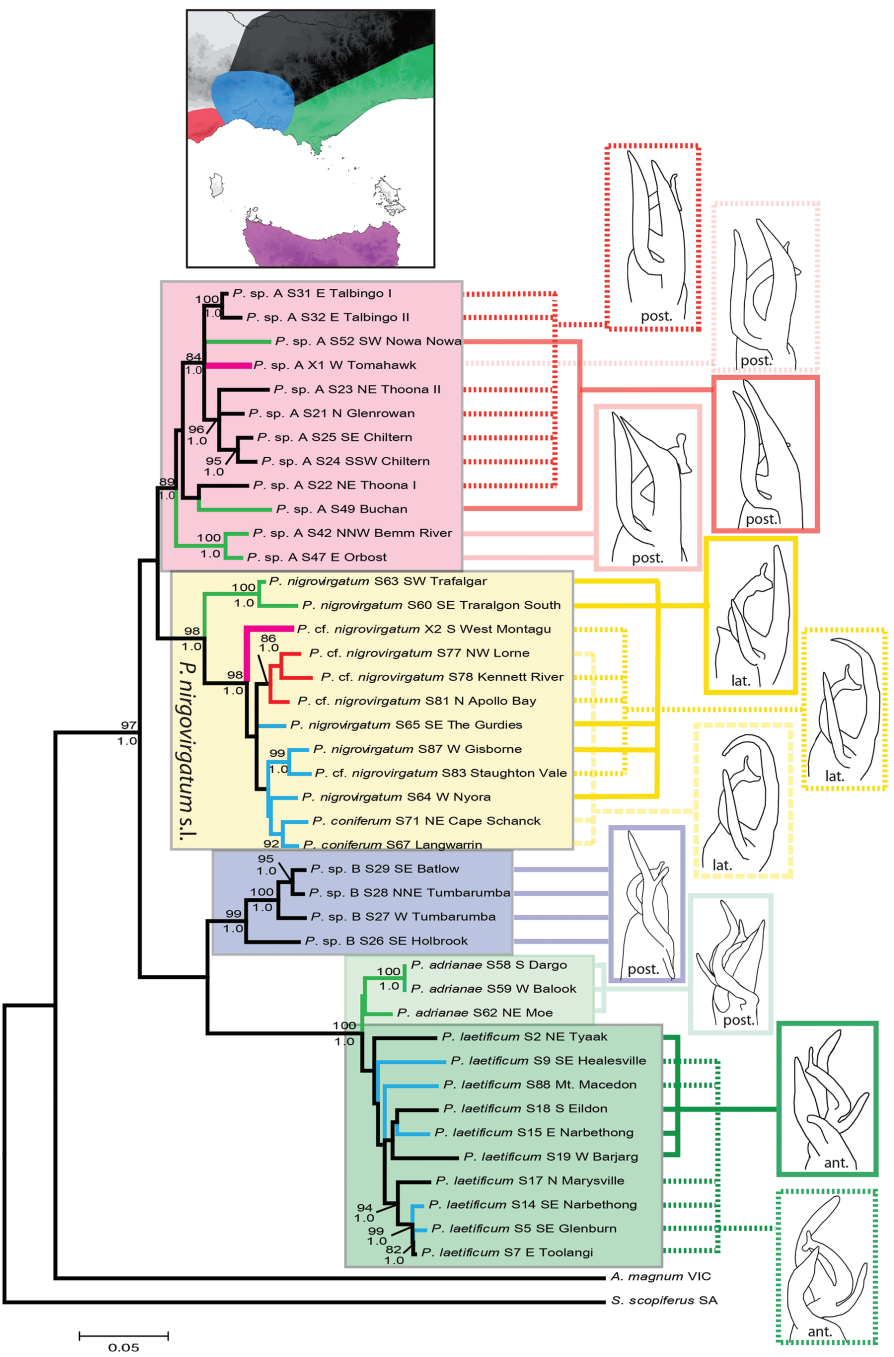
Maximum likelihood tree for the combined mitochondrial COI+16S dataset, 1000 bootstrap replicates, values below 70 not shown. The bootstrap values of ML and posterior probabilities of BI are given above and below the corresponding branches, respectively, for all major clades. Scale bar = substitutions per site. Coloured blocks indicate species groups. Color of branches refers to the major subregions shown in the map, Tasmanian branches thicker. General differences in male gonopod morphology are shown by sketches of the apical region of the right gonopod not drawn to scale. Coloured lines link those analysed specimens that have similar gonopod morphology. Posterior view = post.; lateral view = lat.; anterior view = ant.

Multiple runs of ML and BI converged in trees with the same topology and similar likelihood score so that only the result of the first run is presented. The topology resulting from ML and BI analyses was largely congruent except for the arrangements of several terminal nodes with low support. Thus, results from the ML and BI analyses are shown together based on the ML tree with bootstrap (BP) and posterior probabilities (PP) of the major lineages shown on the corresponding branches with BP values > 70 (Fig. 2).

An appropriate DNA substitution model was determined for 28S under the Bayesian Information Criterion (BIC) in Modeltest implemented in MEGA 6 (Tamura et al. 2011). The lowest Bayesian Information Criterion score (BIC) was obtained for 28S rRNA (BIC 3875.11) with the Tamura 3-parameter model (Tamura 1992).

**Figure 3.**
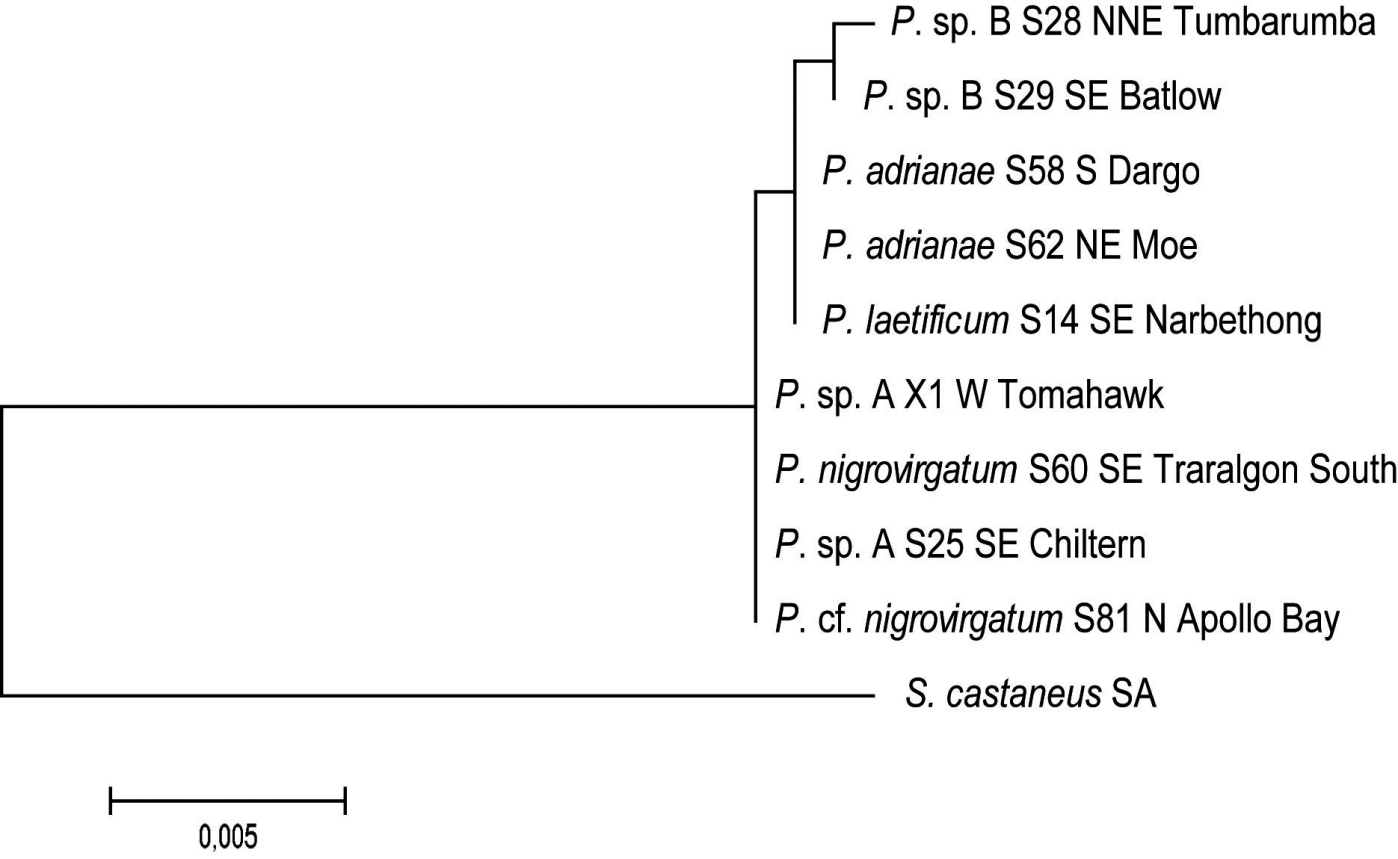
Maximum likelihood tree for the nuclear 28S dataset, 1000 bootstrap replicates, values below 70 not shown.

A phylogenetic hypothesis was inferred for COI+16S and 28S by using the maximum likelihood method conducted in MEGA6 (Tamura et al. 2011). The phylogenetic tree with the highest log likelihood (COI+16S: -7237.4280; 28S: -1831.9238) is shown (Figs 2, 3). Initial trees for the heuristic search were obtained by applying the neighbor-joining method to a matrix of pairwise distances estimated using the Maximum Composite Likelihood (MCL) approach (Tamura et al. 2004). A discrete Gamma distribution was used to model evolutionary rate differences among sites (five categories (+G, parameter = COI+16S: 0.2338)). The bootstrap consensus tree inferred from 1000 replicates (Felsenstein 1985) is here used as the best estimate of the phylogeny of the analyzed taxa (Figs 2, 3).

Mean uncorrected pairwise distances between terminals (transformed into percentages) were determined using MEGA6 (Tamura et al. 2011) and can be found in Suppl. material 3.

## Results

### Phylogenetic and distance analysis

The monophyly of the genus *Pogonosternum* is strongly supported (ML BP = 97; BI PP = 1.0) in the mitochondrial tree and shows five clades within *Pogonosternum*, resembling five species groups (Fig. 2).

One main clade includes three species from the mountainous area east and northeast of Melbourne: the undescribed species *Pogonosternum* sp. B (ML BP = 99; BI PP = 1.0), already mentioned by Car (2010) from New South Wales, *Pogonosternum laetificum* (ML BP= 33; BI PP = 1.0) and *Pogonosternum adrianae* (ML BP = 68; BI PP = 1.0), both not supported, the latter forming a sister clade (ML BP = 100; BI PP = 1.0) to *Pogonosternum* sp. B. The latter two species show moderately large intraspecific distances ranging from 1.1 to 4.6% (*Pogonosternum* sp. B) and 0.1 to 3.0% (*Pogonosternum adrianae*), while *Pogonosternum laetificum* shows high intraspecific distances (0.6–5.5%), even between geographically close (<10 km) populations.

*Pogonosternum nigrovirgatum* sensu lato with a trans-Bass Strait distribution formed a well-supported (ML BP = 89; BI PP = 1.0) sister clade to the new species *Pogonosternum* sp. A (ML BP = 98; BI PP = 1.0) that also has a trans-Bass Strait distribution. *Pogonosternum* sp. A also occurs in New South Wales (Car 2010) and in northeast Tasmania (Mesibov & Churchill 2003). *Pogonosternum nigrovirgatum* s. l. occurs on mainland Australia (Otway Ranges to eastern Victoria) and in northwest Tasmania. *Pogonosternum coniferum* clusters with another form with intermediate gonopods (referred to as Pogonosternum cf. nigrovirgatum in Fig. 2) between *Pogonosternum nigrovirgatum* sensu stricto and *Pogonosternum coniferum*. Both *Pogonosternum nigrovirgatum* s. l. and *Pogonosternum* sp. A show high intraspecific distances ranging from 1.8 to 6.8% within *Pogonosternum nigrovirgatum* s. l. and 1.1 to 5.9% within *Pogonosternum* sp. A.

Within the *Pogonosternum nigrovirgatum* s. l. species-group, the greatest genetic distances were observed between populations in the Strzelecki Ranges (S60, S63; ML BP = 100; BI PP = 1.0) and more western populations, with values ranging from 5.0 to 6.8%. Specimens from the Otway Ranges (S77, S78, S81) all formed a well-supported cluster (ML BP = 86; BI PP = 1.0). The Tasmanian specimen (X2) was distinct from both the Strzelecki Ranges (5.4–6.0%) and central and western Victorian specimens (3.7–3.8%). In the case of *Pogonosternum* sp. A the largest distances (4.2–5.8%) were between the Eastern Gippsland populations (S42, S47; ML BP = 100; BI PP = 1.0) and all other specimens. The status of the northeast Tasmanian specimen is not well resolved; it is closest to a population from Kosciuszko National Park (S31, 3.0%), the two forming a poorly supported sister clade with a specimen from Gippsland (S52; ML BP = 55; BI PP = 0.6).

All species show considerable intraspecific genetic distances and high phylogeographic structure, especially *Pogonosternum laetificum*, and, except in the case of *Pogonosternum adrianae*, no haplotypes are shared between different populations. Additional one to three sequenced specimens from eight sampling sites (S14, S15, S22, S58, S59, S78, S83, S87) always showed the same haplotype in *Pogonosternum* (data not published).

Interspecific distances within the genus *Pogonosternum* are moderately large, varying from 5.5% (*Pogonosternum* sp. A–*Pogonosternum nigrovirgatum* s. l.) to 10.4% (*Pogonosternum nigrovirgatum* s. l.–*Pogonosternum laetificum*), except *Pogonosternum adrianae* to *Pogonosternum laetificum* with only 2.9%.

Owing to the general lack of variability within the nuclear 28S rRNA dataset, the phylogenetic relationships among species were largely unresolved. Distances for 28S rRNA within *Pogonosternum* are very low, with a maximum of three base pair differences noted for *Pogonosternum* sp. B (Fig. 3). Only the two condensed sister clades of *Pogonosternum nigrovirgatum* + *Pogonosternum* sp. A and *Pogonosternum adrianae* + *Pogonosternum laetificum*, as well as *Pogonosternum* sp. B are shown.

### Morphology

In a separate paper (Decker, in preparation), the morphology of the *Pogonosternum* species groups is described in detail and new species are described, based on the specimens used here and from ca 130 additional localities. Here I note briefly that several common morphological features were observed in the gonopods of *Pogonosternum nigrovirgatum* s. l., *Pogonosternum laetificum*, and *Pogonosternum* sp. A: some specimens also showed intermediate states of those features (Fig. 2). It was found, however, when additional material was examined from each population that the morphology of each population was locally stable. It was only in rare cases in the Otway Ranges and NW Tasmania populations that two gonopod morphs occurred in one place.

Surprisingly, gonopod morphology did not appear to agree well with the phylogenetic tree (Fig. 2). Various gonopod forms were distributed with no apparent phylogeographical correlation. Only the species *Pogonosternum adrianae* and *Pogonosternum* sp. B showed stability in both gonopods and some other non-gonopodal characters over their distribution area, even when material from other museum collections was included (Decker, in preparation).

## Discussion

### Phylogenetic analysis

The mitochondrial tree (Fig. 2) shows five main clades, suggesting five species. *Pogonosternum coniferum* clustered within *Pogonosternum nigrovirgatum*, and its taxonomic status needs re-examination (Decker, in preparation).

The 28S tree shows little or only little resolution at the species level (Fig. 3), but was useful in identifying sister clades. This result contrasts with that from a study of the paradoxosomatid genus *Somethus* in South Australia, in which the 28S gene was used successfully for species identification (Decker 2016). Future studies on other Australian Paradoxosomatidae will reveal if 28S is useful as a diagnostic nuclear gene at the species level.

### Morphological variability

With the exception of *Pogonosternum adrianae* and *Pogonosternum* sp. B, *Pogonosternum* species show significant variability in gonopod form, with local morphs occurring throughout each species’ distribution area.

Interestingly, *Pogonosternum adrianae* is morphologically distinct (in size, spiracles, male tibiotarsal brushes and gonopods, female coxal process) from *Pogonosternum laetificum* despite their close genetic distance.

Gonopod variability was also documented for some species of *Somethus* in South Australia (Decker 2016) and *Stygiochiropus* Humphreys & Shear, 1993 from Western Australia (Humphreys and Shear 1993). Another good example of variability is seen in the trans-Bass Strait (eastern Victoria, NE Tasmania) paradoxosomatid millipede, *Dicranogonus pix*: while this species shows only slight variability in gonopods there is marked variation in the development of their paranota. Individuals with no paranota are separated from those with keels by a gap between the Kent and Furneaux Groups of islands (Mesibov 2014).

This study has shown that in the area of southern and southeast Australia, there are at least two genera, *Pogonosternum* and *Somethus* (Decker 2016), which both show variability in morphology and genetics. Poor sampling and too few specimens could lead to incorrect conclusions and unnecessary multiple species descriptions.

### Multiple glacial refugia in southeastern Australia

The results indicate that there is high intraspecific genetic divergence, with high genetic distances and haplotype diversity in the mitochondrial genes between populations of *Pogonosternum*, even those adjacent to each other. The *Pogonosternum laetificum* clade, which has been sampled extensively in the Central Highlands, shows particularly high intraspecific genetic differences (mean genetic distance of 3.9%), apparently without corresponding geographic patterning, or morphological variation (Decker, in preparation).

The phylogenetic patterns with high intraspecific divergence, high genetic distances, and haplotype diversity with unique local haplotypes, resulting in long branches, shown by *Pogonosternum*, indicate multiple Pleistocene refugia according to Byrne (2008). These refugia provided suitably moist habitats in which species could persist during the dry, cold climate cycles of the Pleistocene period in southern Australia, while glaciation was limited to the alpine areas of the Great Dividing Range and Tasmania (Barrows et al. 2002). Moderate to high genetic diversity prior to these cycles can be assumed for poorly dispersing millipedes, through isolation by distance, and it is likely that populations were isolated within refugia, leading to further genetic diversification. In contrast, contractions to one or few major refugia during cold, arid periods would result in a low genetic diversity, few divergent lineages and low haplotype diversity, with few haplotypes in areas of postglacial recolonisation (Byrne 2008).

The phylogenetic patterns shown by *Pogonosternum* suggest that in Victoria and New South Wales there were large areas with multiple local refugia during the Pleistocene. No region in the study area on mainland Australia showed results which indicate rapid postglacial resettlement of *Pogonosternum*.

Evidence for multiple glacial refugia was also identified in the spirostreptidan millipede *Atelomastix bamfordi* Edward & Harvey, 2010 in Western Australia (Nistelberger et al. 2014) and for some species of *Somethus* in South Australia (Decker 2016). Similar phylogeographic patterns seem to occur in other soil invertebrates with limited dispersal capacities in southern Australia, for example flatworms (Sunnucks et al. 2006) and springtails (Garrick et al. 2004).

Endo et al. (2014) have suggested, however, that glacial periods have had less of an impact on the distribution and genetic diversity of invertebrate groups (Coleoptera, Orthoptera, Collembola, Diplopoda) in the Australian Alps than they have in alpine systems in the Northern Hemisphere.

However, further studies on genetic and morphological variability on a finer geographical scale could lead to a better understanding of the pattern and impact of isolation in multiple glacial refugia during the Pleistocene, also as an evolutionary driving force for morphological variability in some species.

### Gippsland phylogeography

There is a notable high genetic distance gap within *Pogonosternum nigrovirgatum* sensu lato between specimens from the Strzelecki Ranges (S60, S63), West Gippsland, and those sampled in the central and western regions in Victoria, but some specimens of adjacent populations from the latter (S64, S65) were morphologically indistinguishable from specimens from the Strzelecki Ranges. A similar genetic gap was observed in *Pogonosternum* sp. A for the populations in Eastern Gippsland east of Orbost (S42, S47) and all other populations. These two cases indicate that these areas may have been isolated for long periods from neighboring regions, possibly before the Pleistocene, perhaps during a marine incursion in the Gippsland Basin and other parts of southeast Australia close to the Miocene–Pliocene boundary (Dickinson et al. 2002).

### Trans-Bass Strait distribution

The genus *Pogonosternum* shows a trans-Bass Strait distribution and most likely originated in mainland southeast Australia, since the highest species diversity is found on the mainland and the two Tasmanian branches occupy only very subordinate positions on the tree (Fig. 2). Tasmanian populations of this genus are restricted to the northeast and northwest corners of the Tasmanian mainland and neighboring islands, and presumably dispersed from Victoria when it was largely connected with Tasmania during the Pleistocene (Lambeck and Chappell 2001). Mitochondrial data suggest that the sequenced population of *Pogonosternum nigrovirgatum* s. l. in northwest Tasmania was most likely derived from one in central Victoria or the Otway Ranges. While the results for *Pogonosternum* sp. A from northeast Tasmania do not show a close relationship to coastal Victorian populations, analysis of 16S (data not included here) including sequences from two other localities in the western part of East Gippsland showed the Tasmanian specimen clustering with the latter. This indicates that the settlement of Tasmania by this species started in the Gippsland region. A remarkably similar distribution to that of *Pogonosternum* sp. A across Bass Strait is also known for the paradoxosomatid millipedes *Dicranogonus pix* and *Notodesmus scotius* (Mesibov 2014).

Further studies using more sampling localities in Tasmania and its islands could indicate points of origin in Victoria and the timing of millipede settlement of Tasmania.

## Supplementary materials

Supplementary material 1 Full data of sequenced specimens Data type: Tab-delimited text file Explanation note: Full details of sequenced specimens, including locality, date, collector, collection number and coordinates. This dataset is made available under the Open Database License (http://opendatacommons.org/licenses/odbl/1.0/). The Open Database License (ODbL) is a license agreement intended to allow users to freely share, modify, and use this Dataset while maintaining this same freedom for others, provided that the original source and author(s) are credited.Peter Decker

Supplementary material 2 Alignment of combined dataset Data type: FASTA file Explanation note: Alignment of the combined COI mtDNA and 16S rRNA dataset This dataset is made available under the Open Database License (http://opendatacommons.org/licenses/odbl/1.0/). The Open Database License (ODbL) is a license agreement intended to allow users to freely share, modify, and use this Dataset while maintaining this same freedom for others, provided that the original source and author(s) are credited.Peter Decker

Supplementary material 3 P-distances of combined COI and 16S dataset Data type: CSV File Explanation note: Mean uncorrected pairwise distances between terminals (transformed into percentages) of the combined COI mtDNA and 16S rRNA dataset. This dataset is made available under the Open Database License (http://opendatacommons.org/licenses/odbl/1.0/). The Open Database License (ODbL) is a license agreement intended to allow users to freely share, modify, and use this Dataset while maintaining this same freedom for others, provided that the original source and author(s) are credited.Peter Decker
